# Improving the Methodology for Identifying Mild Cognitive Impairment in Intellectually High-Functioning Adults Using the NIH Toolbox Cognition Battery

**DOI:** 10.3389/fpsyg.2021.724888

**Published:** 2021-09-08

**Authors:** Grant L. Iverson, Justin E. Karr

**Affiliations:** ^1^Department of Physical Medicine and Rehabilitation, Harvard Medical School, Boston, MA, United States; ^2^Spaulding Rehabilitation Hospital and Spaulding Research Institute, Charlestown, MA, United States; ^3^Home Base, A Red Sox Foundation and Massachusetts General Hospital Program, Charlestown, MA, United States; ^4^Department of Psychology, University of Kentucky, Lexington, KY, United States

**Keywords:** cognition, neuropsychological tests, psychometrics, cognitive dysfunction, NIH Toolbox

## Abstract

**Objective:** Low scores on neuropsychological tests are considered objective evidence of mild cognitive impairment. In clinical practice and research, it can be challenging to identify a cognitive deficit or mild cognitive impairment in high-functioning people because they are much less likely to obtain low test scores. This study was designed to improve the methodology for identifying mild cognitive impairment in adults who have above average or superior intellectual abilities.

**Method:** Participants completed the National Institutes of Health Toolbox for the Assessment of Neurological and Behavioral Function Cognition Battery (NIHTB-CB). The sample included 384 adults between the ages of 20 and 85 who had completed either a 4-year college degree or who scored in the above average, superior, or very superior range on a measure of intellectual functioning, the Crystallized Composite score. Algorithms were developed, based on the absence of high scores and the presence of low scores, for identifying mild cognitive impairment.

**Results:** Base rate tables for the presence of low scores and the absence of high scores are provided. The base rate for people with high average crystalized ability obtaining any one of the following, 5 scores <63rd percentile, or 4+ scores <50th percentile, or 3+ scores ≤ 25th percentile, or 2+ scores ≤ 16th percentile, is 15.5%.

**Conclusions:** Algorithms were developed for identifying cognitive weakness or impairment in high-functioning people. Research is needed to test them in clinical groups, and to assess their association with clinical risk factors for cognitive decline and biomarkers of acquired neurological or neurodegenerative diseases.

## Introduction

Deficit measurement is the *sine qua non* of clinical neuropsychology. Low scores on neuropsychological tests are used to define a cognitive deficit or mild cognitive impairment (Heaton et al., 1991, 2004; Reitan and Wolfson, 1993; Petersen et al., 1999; Dubois et al., 2007). However, if many tests are administered, most healthy adults and older adults will obtain one or more low scores (Palmer et al., 1998; Axelrod and Wall, 2007; Schretlen et al., 2008; Binder et al., 2009; Brooks et al., 2009a, 2010, 2011). In fact, for healthy adults of average intelligence, with no known form of cognitive impairment, it is common to obtain up to 20–25% of their test scores, across a battery of tests, at or below one standard deviation (SD) from the mean (Brooks et al., 2009a, 2011). Even within a single cognitive domain, such as memory or executive function, it is common for healthy children, adults, and older adults to obtain one or more low test scores (Brooks et al., 2008, 2009b,c; Karr et al., 2017, 2018; Cook et al., 2019). This makes it challenging to accurately identify mild cognitive impairment (Petersen et al., 1999; Albert et al., 2011) or mild neurocognitive disorder, based on the Diagnostic and Statistical Manual of Mental Disorders, Fifth Edition (DSM-5) criteria (American Psychiatric Association, 2013), because these diagnostic criteria require test performance that is greater than one SD from the mean, but the criteria do not specify exactly *how* that is determined—such as whether one or more test scores in this range are required.

There is a strong association between higher intelligence and higher neuropsychological test scores (Warner et al., 1987; Tremont et al., 1998; Horton, 1999; Steinberg et al., 2005a,b). In clinical practice and research, it can be challenging to identify a cognitive deficit or mild cognitive impairment in high-functioning people because they are much less likely to obtain low test scores (Brooks et al., 2009a, 2011), and a much greater change in functioning needs to occur, as a result of a neurological disease, before they perform one or more SDs below the normative mean. Therefore, in some high-functioning people, it might be the *absence of high scores*, more so than the presence of low scores, that reveals their cognitive decline.

This study was designed to improve the methodology for identifying mild cognitive impairment in adults who have above average or superior intellectual abilities. The adult standardization sample for the National Institutes of Health Toolbox for the Assessment of Neurological and Behavioral Function (Gershon et al., 2010, 2013) Cognition Battery (NIHTB-CB) (Weintraub et al., 2013) was used to develop algorithms for defining cognitive impairment. This 30-min battery is comprised of seven tests measuring attention, working memory, language, processing speed, and executive functioning. The algorithms incorporate concepts from recent studies illustrating that the *absence of high scores* is uncommon in high-functioning people (Karr and Iverson, 2020; Karr et al., 2020). For example, considering the five fluid scores from the NIH Toolbox, only 17–19% of adults with above average or superior intelligence will have no above average fluid cognition scores (Karr and Iverson, 2020). Therefore, in high-functioning people, cognitive deficits might be reflected by the *presence of low scores, the absence of high scores, or both*. This study will combine criteria relating to both low scores (Holdnack et al., 2017) and high scores (Karr and Iverson, 2020) to propose a new method for identifying mild cognitive impairment in adults with above average or superior intellectual abilities.

## Method

### Participants

The normative sample for the NIHTB-CB (Gershon, 2016) includes 1,021 adult participants between the ages of 20 and 89, of whom 843 completed all seven tests. Previously published studies reported low and high score base rates using the entire NIHTB-CB normative sample, including those with pre-existing neurodevelopmental, psychiatric, substance use, and neurological disorders (Holdnack et al., 2017; Karr and Iverson, 2020), whereas these base rates were re-calculated for the current study including only those participants who did not report *any* of these pre-existing conditions. Participants were excluded from analysis if they reported (a) a pre-existing neurodevelopmental disorder, including a specific learning disability (*n* = 9), attention-deficit/hyperactivity disorder (*n* = 16), Asperger's syndrome (*n* = 1), or a developmental delay (*n* = 1); (b) a psychiatric or substance use disorder, including a serious emotional disturbance (*n* = 8), bipolar disorder or schizophrenia (*n* = 8), depression or anxiety (*n* = 92), alcohol abuse (*n* = 3), drug abuse (*n* = 5), or a hospitalization due to emotional problems (*n* = 7); or (c) a neurological disorder, including epilepsy or seizures (*n* = 5), traumatic brain injury (*n* = 1), multiple sclerosis (*n* = 1), a stroke or transient ischemic attack (*n* = 7), or a history of brain surgery (*n* = 8). Some participants had more than one of these conditions. This resulted in a final sample of 730 participants (age: *M* = 47.4 years, *SD* = 17.6, range: 18 to 85; 35.6% men, 64.4% women; education: *M* = 14.2 years, *SD* = 2.5). The racial and ethnic breakdown of the sample was as follows: 63.1% White, 17.7% African American, 9.7% Latinx, 4.0% Asian or Pacific Islander, 1.6% Multiracial, 1.0% Native American, 1.0% Afro-Latinx, and 1.9% not provided. A subsample of these participants (*n* = 687) had sufficient data to calculate demographic-adjusted T scores (age: *M* = 47.8 years, *SD* = 17.6, range: 18–85; 35.7% men, 64.3% women; education: *M* = 14.3 years, *SD* = 2.5). The racial and ethnic breakdown of that sample was as follows: 67.1% White, 18.6% African American, 10.3% Latinx, and 3.9% Asian.

Of the samples described above, 394 met at least one of the following criteria: (a) having 16 or more years of education, (b) obtaining an age-adjusted Crystallized Composite Standard Score of 110 or greater, or (c) obtaining a demographic-adjusted Crystallized Composite T score of 57 or greater. The average age of this sample was 47.0 years (*SD* = 17.2) and the sample includes 38.1% men and 61.9% women. Their average education was 15.7 years (*SD* = 2.2). The racial and ethnic breakdown of the sample was as follows: 66.8% White, 16.5% African American, 10.2% Latinx, 4.6% Asian, 0.8% Multiracial, 0.3% Afro-Latinx, and 0.3% Native American. This sample was used to prepare algorithms for identifying cognitive impairment in high-functioning people.

### Measures

The NIHTB-CB includes seven tests, from which three composites are derived by averaging normalized scores: the Total Composite, the Crystallized Composite, and the Fluid Composite. The Total Composite is derived from all seven tests, whereas the other composites are derived from a subset of scores. The Crystallized Composite is composed of two tests: Picture Vocabulary and Oral Reading Recognition. These tests have been shown to correlate with tests of word reading and receptive vocabulary (Gershon et al., 2014), which correlate with intelligence and are commonly used as estimates of premorbid intellectual functioning. In this study, the Crystallized Composite score serves as our estimate of a person's level of intelligence. The Fluid Composite is composed of five tests: a measure of working memory, the List Sorting Working Memory (Tulsky et al., 2014); a measure of episodic memory, Picture Sequence Memory (Dikmen et al., 2014); a measure of processing speed, Pattern Comparison Processing Speed (Carlozzi et al., 2014); and measures of inhibitory control and cognitive flexibility, Flanker Inhibitory Control and Attention and Dimensional Change Card Sort, respectively (Zelazo et al., 2014). Detailed descriptions of each test are reported elsewhere (Holdnack et al., 2017).

### Procedures

The normative data for the English-language NIHTB-CB was collected as part of a national norming study involving recruitment of a sample of children, adolescents, and adults representative of the U.S. population per 2010 U.S. Census data (Beaumont et al., 2013). The adult sample consisted of community-dwelling adults who were capable of following test instructions in English and provided informed consent prior to participation. The fully deidentified normative data are publicly available for download for secondary analysis (Gershon, 2016). The secondary analyses of these deidentified data were deemed not human subjects research and were approved by the Partners Human Research Committee (Protocol #: 2020P000504).

### Statistical Analyses

For the NIHTB-CB, age-adjusted scores are standardized as Standard Scores (SS; *M* = 100, *SD* = 15) and the demographic-adjusted scores are standardized as T scores (*M* = 50, *SD* = 10) with adjustments for age, gender, education, and race/ethnicity (Casaletto et al., 2015). The following cutoffs were used to define performances at or below specific percentiles: ≤ 25th percentile (SS ≤ 90 or T ≤ 43), ≤ 16th percentile (SS ≤ 85 or T ≤ 40), ≤ 9th percentile (SS ≤ 80 or T ≤ 36), ≤ 5th percentile (SS ≤ 76 or T ≤ 34), and ≤ 2nd percentile (SS ≤ 70 or T ≤ 30). Of note, no whole number T score corresponds to the exact 9th percentile, and a T ≤ 36 was selected because it corresponds to the lowest whole number T score typically interpreted as borderline or unusually low in clinical practice. The following cutoffs were used for defining scores at or above certain cutoffs: ≥50th percentile (SS≥100 or T≥50), ≥63rd percentile (SS≥105 or T≥53), ≥75th percentile (SS≥110 or T≥57), ≥84th percentile (SS≥115 or T≥60), ≥91st percentile (SS≥120 or T≥64), ≥95th percentile (SS≥124 or T≥66), and ≥98th percentile (SS≥130 or T≥70). Of note, a T score of 53 was selected as the closest whole number T score to the 63rd percentile and a T score of 64 was selected as the closest whole number T score to the 91st percentile, but they align more closely with the 62nd percentile and 92nd percentile, respectively. The percentile cutoffs for defining low and high scores are consistent with previous research on multivariate base rates using the NIHTB-CB (Holdnack et al., 2017; Karr and Iverson, 2020). Although all the above cutoffs, collectively, are described as high score base rates, performances falling ≥50th and ≥63rd percentiles are not typically interpreted as *high* in clinical practice, but they are useful for determining whether an *absence* of scores above these cutoffs is unusual for high functioning individuals, and potentially indicative of cognitive impairment.

## Results

### Base Rates of Low Scores

The base rates of low scores on the NIHTB-CB, for the total sample and stratified by years of education and Crystallized Composite, are presented in Table 1. Base rates are presented for several different cutoff scores, including ≤ 25th, ≤ 16th, ≤ 9th, ≤ 5th, and ≤ 2nd percentiles for both age-adjusted normative scores and demographic-adjusted normative scores. Using age-adjusted norms, people with higher levels of education and above average or superior scores on the Crystallized Composite, obtained fewer low scores. For example, using the 16th percentile as the cutoff for a low score, the base rates of having one or more low scores, by subgroup, were as follows: education = 12 years, 49.2%; education = 16 or more years, 36.0%; crystallized composite = 110–119, 22.7%; and crystallized composite = 120 or greater, 19.2%.

**Table 1:** Base rates of low scores on NIHTB-CB fluid measures in the normative sample without pre-existing neurobehavioral, psychiatric, substance use, or neurological conditions, with stratifications by education and crystallized ability level.

	**Age Norms**	**Age norms by education level**				**Age norms by crystallized composite**					**Demo. Norms**	**Demo. norms by crystallized composite**				
**Number of low scores**		** <12**	**12**	**13–15**	**≥16**	**≤89**	**90–99**	**100–109**	**110–119**	**≥120**		**≤43**	**44–49**	**50–56**	**57–63**	**≥64**
Sample size	730	68	195	170	292	179	179	189	110	73	687	183	146	180	122	56
**≤25th percentile**																
5 low scores	2.6	2.9	3.6	2.9	1.7	7.3	2.2	1.1	–	–	2.2	5.5	2.7	0.6	–	–
4 or more	8.2	17.6	8.2	8.2	6.2	20.7	8.9	3.7	–	–	7.1	16.4	8.9	1.7	2.5	–
3 or more	17.7	33.8	20.5	16.5	13.0	41.3	17.9	9.0	3.6	2.7	14.6	31.7	15.8	6.7	3.3	5.4
2 or more	35.5	51.5	40.0	34.7	29.1	60.9	40.2	28.0	17.3	8.2	33.2	58.5	33.6	25.0	14.8	16.1
1 or more	62.3	80.9	66.2	65.3	54.1	86.0	64.2	56.1	47.3	38.4	62.0	79.8	65.1	60.0	43.4	42.9
No low scores	37.7	19.1	33.8	34.7	45.9	14.0	35.8	43.9	52.7	61.6	38.0	20.2	34.9	40.0	56.6	57.1
**≤16th percentile**																
5 low scores	0.5	1.5	0.5	1.2	–	2.2	–	–	–	–	0.6	2.2	–	–	–	–
4 or more	3.2	7.4	3.1	2.9	2.4	11.7	0.6	0.5	–	–	3.9	9.8	4.1	0.6	1.6	–
3 or more	8.9	16.2	9.2	8.2	7.5	21.8	8.9	3.7	1.8	1.4	8.9	21.3	8.2	3.9	2.5	–
2 or more	20.1	27.9	24.1	19.4	16.4	38.0	25.1	13.2	5.5	4.1	20.7	37.7	21.9	13.3	9.8	8.9
1 or more	42.9	57.4	49.2	42.4	36.0	68.7	46.9	35.4	22.7	19.2	45.4	69.4	45.9	38.9	23.8	33.9
No low scores	57.1	42.6	50.8	57.6	64.0	31.3	53.1	64.6	77.3	80.8	54.6	30.6	54.1	61.1	76.2	66.1
**≤9th percentile**																
5 low scores	0.1	1.5	–	–	–	0.6	–	–	–	–	–	–	–	–	–	–
4 or more	0.8	4.4	0.5	0.6	0.3	3.4	–	–	–	–	0.9	3.3	–	–	–	–
3 or more	3.4	8.8	3.1	4.1	2.1	11.2	2.2	0.5	–	–	2.6	7.1	2.1	–	1.6	–
2 or more	10.5	19.1	12.3	12.4	6.5	25.7	11.7	4.8	0.9	–	8.9	20.2	8.9	3.3	4.1	–
1 or more	28.1	39.7	32.8	28.2	22.6	49.7	29.1	22.8	10.9	12.3	27.5	48.1	28.1	20.0	11.5	17.9
No low scores	71.9	60.3	67.2	71.8	77.4	50.3	70.9	77.2	89.1	87.7	72.5	51.9	71.9	80.0	88.5	82.1
**≤5th percentile**																
4 low scores	0.3	1.5	0.5	–	–	1.1	–	–	–	–	0.6	2.2	–	–	–	–
3 or more	1.5	4.4	1.0	1.8	1.0	6.1	–	–	–	–	1.6	4.9	1.4	–	–	–
2 or more	4.8	10.3	5.6	4.1	3.4	14.5	5.0	–	–	–	4.7	12.6	4.1	1.1	0.8	–
1 or more	18.9	32.4	20.5	19.4	14.7	38.0	16.8	14.3	5.5	9.6	20.5	38.3	16.4	17.2	8.2	10.7
No low scores	81.1	67.6	79.5	80.6	85.3	62.0	83.2	85.7	94.5	90.4	79.5	61.7	83.6	82.8	91.8	89.3
**≤2nd percentile**																
4 low scores	–	–	–	–	–	–	–	–	–	–	0.1	0.5	–	–	–	–
3 or more	0.4	2.9	–	0.6	–	1.7	–	–	–	–	0.4	1.6	–	–	–	–
2 or more	2.1	5.9	2.1	1.8	1.4	6.7	1.7	–	–	–	2.2	6.6	1.4	–	0.8	–
1 or more	8.8	20.6	8.2	9.4	6.2	19.6	8.9	4.2	1.8	4.1	9.0	17.5	6.8	7.2	3.3	5.4
No low scores	91.2	79.4	91.8	90.6	93.8	80.4	91.1	95.8	98.2	95.9	91.0	82.5	93.2	92.8	96.7	94.6

*Demo., Demographic-adjusted; NIHTB-CB, National Institutes of Health Toolbox for the Assessment of Neurological and Behavioral Function Cognition Battery. All values represent cumulative percentages except for the rows labeled “No low scores,” which provide the percentage of the normative sample with no scores falling below the low score cut-offs. Age norms are provided as age-adjusted standard scores (M = 100, SD-15) and demographic-adjusted norms are provided as T scores (M = 50, SD = 10) adjusted for age, sex, education level, and race/ethnicity. The NIHTB-CB includes five tests of fluid abilities: Flanker Inhibitory Control and Attention Test, Picture Sequence Memory Test, List Sorting Working Memory Test, Dimensional Change Card Sort Test, and Pattern Comparison Processing Speed Test. Two tests measure crystallized abilities, from which the crystallized composite is calculated: Picture Vocabulary Test and Oral Reading Recognition Test*.

### Base Rates of High Scores

The base rates of high scores on the NIHTB-CB, for the total sample and stratified by education and level of intellectual functioning, are presented in Table 2. Base rates are presented for several different cutoff scores, including ≥50th, ≥63rd, ≥75th, ≥84th, ≥91st, ≥95th, and ≥98th percentiles for both age-adjusted normative scores and demographic-adjusted normative scores. Using age-adjusted norms, people with higher levels of education and above average or superior scores on the Crystallized Composite obtained more high scores. For example, using the 84th percentile as the cutoff for a high score, the base rates of having two or more high scores, by subgroup, were as follows: education = 12 years, 20.0%; education = 16 or more years, 27.1%; crystallized composite = 110-119, 33.6%; and crystallized composite = 120 or greater, 45.2%. Using the 95th percentile as the cutoff for a high score, the base rates of having one or more high scores, by subgroup, were as follows: education = 12 years, 19.5%; education = 16 or more years, 29.1%; crystallized composite = 110-119, 34.5%; and crystallized composite = 120 or greater, 42.5%.

**Table 2:** Base rates of high scores on NIHTB-CB fluid measures in the normative sample without pre-existing neurobehavioral, psychiatric, substance use, or neurological conditions, with stratifications by education and crystallized ability level.

	**Age Norms**	**Age norms by education level**				**Age norms by crystallized composite**					**Demo. Norms**	**Demo. norms by crystallized composite**				
**Number of high scores**		**<12**	**12**	**13–15**	**≥16**	**≤89**	**90–99**	**100–109**	**110–119**	**≥120**		**≤43**	**44–49**	**50–56**	**57–63**	**≥64**
**≥50th percentile**																
5 high scores	11.9	4.4	9.2	7.6	17.8	1.7	5.6	15.9	20.9	28.8	11.6	3.3	8.9	14.4	16.4	26.8
4 or more	33.6	19.1	29.7	32.4	40.1	13.4	27.4	36.5	48.2	68.5	32.9	14.8	29.5	33.9	50.0	60.7
3 or more	53.8	35.3	48.7	54.1	61.3	30.2	51.4	58.2	72.7	78.1	56.2	37.7	54.1	58.3	71.3	82.1
2 or more	73.3	55.9	69.7	73.5	79.5	50.3	68.2	81.5	90.0	95.9	74.8	54.6	71.9	81.1	90.2	94.6
1 or more	90.7	88.2	89.2	90.0	92.5	78.8	88.3	95.8	99.1	100	90.1	79.8	85.6	95.6	98.4	100
No high scores	9.3	11.8	10.8	10.0	7.5	21.2	11.7	4.2	0.9	0	9.9	20.2	14.4	4.4	1.6	0
**≥63rd percentile**																
5 high scores	5.9	1.5	3.6	2.9	10.3	0.6	2.2	7.4	11.8	15.1	6.8	0.5	3.4	9.4	10.7	19.6
4 or more	16.2	10.3	12.3	14.1	21.2	2.8	10.6	17.5	28.2	41.1	20.1	6.6	15.1	21.1	32.8	46.4
3 or more	36.4	19.1	32.8	35.3	43.8	13.4	32.4	41.8	50.0	68.5	37.8	18.6	31.5	43.3	54.9	62.5
2 or more	56.4	38.2	50.8	56.5	64.4	31.8	51.4	61.9	76.4	84.9	58.1	41.5	54.1	63.3	69.7	80.4
1 or more	79.0	70.6	75.9	78.8	82.9	62.0	73.2	86.2	93.6	94.5	83.1	72.1	77.4	87.8	91.8	100
No high scores	21.0	29.4	24.1	21.2	17.1	38.0	26.8	13.8	6.4	5.5	16.9	27.9	22.6	12.2	8.2	0
**≥75th percentile**																
5 high scores	2.2	–	1.0	1.2	4.1	–	–	2.1	6.4	6.8	2.3	–	0.7	3.9	3.3	7.1
4 or more	7.4	2.9	5.6	5.9	10.6	1.1	3.9	8.5	13.6	19.2	7.7	1.6	4.8	8.9	11.5	23.2
3 or more	19.3	7.4	17.4	20.6	22.9	6.1	15.1	19.6	29.1	46.6	20.4	6.0	17.1	22.2	29.5	50.0
2 or more	38.8	20.6	37.4	38.2	44.2	15.6	34.1	45.5	54.5	65.8	39.7	19.7	37.0	47.2	51.6	62.5
1 or more	66.3	51.5	62.6	67.6	70.9	43.6	63.7	75.7	79.1	84.9	66.5	51.9	62.3	71.1	77.0	87.5
No high scores	33.7	48.5	37.4	32.4	29.1	56.4	36.3	24.3	20.9	15.1	33.5	48.1	37.7	28.9	23.0	12.5
**≥84th percentile**																
5 high scores	0.4	–	–	–	1.0	–	–	–	1.8	1.4	0.6	–	–	1.1	0.8	1.8
4 or more	2.9	–	2.1	2.4	4.5	–	2.2	2.6	7.3	5.5	2.9	1.1	0.7	5.0	2.5	8.9
3 or more	8.4	2.9	7.2	7.6	11.0	2.2	5.0	9.0	12.7	23.3	8.7	1.6	6.8	12.2	10.7	21.4
2 or more	21.9	10.3	20.0	20.6	27.1	7.8	15.1	25.9	33.6	45.2	24.2	10.4	23.3	25.6	32.0	50.0
1 or more	48.6	29.4	48.2	47.6	53.8	24.0	45.3	57.7	58.2	79.5	52.7	37.7	43.8	59.4	66.4	73.2
No high scores	51.4	70.6	51.8	52.4	46.2	76.0	54.7	42.3	41.8	20.5	47.3	62.3	56.2	40.6	33.6	26.8
**≥91st percentile**																
5 high scores	0.1	–	–	–	0.3	–	–	–	0.9	–	–	–	–	–	–	–
4 or more	1.1	–	1.0	1.2	1.4	–	1.1	0.5	3.6	1.4	0.7	–	–	1.1	0.8	3.6
3 or more	3.0	–	2.1	3.5	4.1	0.6	1.7	2.6	10.0	2.7	2.9	1.1	0.7	4.4	4.1	7.1
2 or more	10.3	2.9	9.7	10.0	12.7	3.9	7.3	9.0	20.0	21.9	11.4	5.5	8.9	15.6	9.0	28.6
1 or more	32.7	17.6	28.2	31.2	40.1	14.0	30.2	37.0	43.6	57.5	36.7	26.2	30.8	40.0	44.3	58.9
No high scores	67.3	82.4	71.8	68.8	59.9	86.0	69.8	63.0	56.4	42.5	63.3	73.8	69.2	60.0	55.7	41.1
**≥95th percentile**																
4 high scores	0.4	–	0.5	0.6	0.3	–	–	–	2.7	–	0.3	–	–	–	–	3.6
3 or more	1.4	–	0.5	2.4	1.7	–	0.6	1.1	5.5	1.4	1.2	0.5	–	1.1	2.5	3.6
2 or more	5.2	1.5	4.6	4.7	6.8	1.7	3.4	4.2	10.9	12.3	4.8	1.6	2.7	3.9	7.4	17.9
1 or more	23.0	13.2	19.5	20.6	29.1	7.8	21.2	24.9	34.5	42.5	22.3	11.5	19.9	27.2	27.0	37.5
No high scores	77.0	86.8	80.5	79.4	70.9	92.2	78.8	75.1	65.5	57.5	77.7	88.5	80.1	72.8	73.0	62.5
**≥98th percentile**																
3 high scores	0.3	–	–	–	0.7	–	–	–	1.8	–	0.3	–	–	0.6	–	1.8
2 or more	1.6	–	1.5	1.8	2.1	–	1.7	0.5	7.3	–	1.7	0.5	0.7	2.8	1.6	5.4
1 or more	10.1	4.4	9.7	8.8	12.3	2.2	9.5	7.9	20.9	20.5	9.2	4.4	5.5	10.6	13.9	19.6
No high scores	89.9	95.6	90.3	91.2	87.7	97.8	90.5	92.1	79.1	79.5	90.8	95.6	94.5	89.4	86.1	80.4

*Demo., Demographic-adjusted; NIHTB-CB, National Institutes of Health Toolbox for the Assessment of Neurological and Behavioral Function Cognition Battery. All values represent cumulative percentages except for the rows labeled “No high scores,” which provide the percentage of the normative sample with no scores falling above the high score cut-offs. Age norms are provided as age-adjusted standard scores (M = 100, SD-15) and demographic-adjusted norms are provided as T scores (M = 50, SD = 10) adjusted for age, sex, education level, and race/ethnicity. The NIHTB-CB includes five tests of fluid abilities: Flanker Inhibitory Control and Attention Test, Picture Sequence Memory Test, List Sorting Working Memory Test, Dimensional Change Card Sort Test, and Pattern Comparison Processing Speed Test. Two tests measure crystallized abilities, from which the crystallized composite is calculated: Picture Vocabulary Test and Oral Reading Recognition Test*.

As seen in Table 2, it is uncommon to obtain no scores ≥50th percentile or ≥63rd percentile, which occurred in only 9.3 and 21.0% of the total sample, respectively, using age-adjusted norms. The absence of scores at or above these cutoffs was very uncommon in individuals of high average crystallized ability, occurring in only 0.9 and 6.4%, respectively, using age-adjusted norms. Using demographic-adjusted norms, 91.8% of those with high average crystallized ability and 100% of individuals with superior crystallized ability obtained at least 1 score ≥63rd percentile.

### Algorithms for Identifying Cognitive Impairment

The algorithms in Table 3 rely on age-adjusted normative scores. We have computed the base rate of each component of the algorithm separately, and then the base rate for the entire algorithm. For Algorithm A, for example, the base rate for people with high average crystalized ability obtaining *any one* of the following, 5 scores <63rd percentile, or 4+ scores <50th percentile, or 3+ scores ≤ 25th percentile, or 2+ scores ≤ 16th percentile, is 15.5%. As such, having a performance patten on the NIHTB-CB consistent with that algorithm would correspond to 1 SD below the mean for people with high average intellectual abilities. For Algorithm D, the base rate for people with university degrees obtaining 4+ scores ≤ 25th percentile *or* 2+ scores ≤ 5th percentile is 7.5%. As such, a performance consistent with that algorithm is ~1.5 SDs below the mean for people with university degrees.

**Table 3:** Primary algorithms for identifying cognitive impairment in high functioning adults on the NIH toolbox cognition battery using age-adjusted normative scores.

	**High average**	**Superior**	**4-Year College/**
	**(SS = 110–119)**	**(SS ≥ 120)**	**University Degree**
	**(*n* = 110)**	**(*n* = 73)**	**(*n* = 292)**
**Algorithms and criteria**	**Base rate**	**Base rate**	**Base rate**
**Algorithm A**	**15.5%**	**11.0%**	**28.8%**
5 scores <63rd percentile, or	6.4%	5.5%	17.1%
4+ scores <50th percentile, or	10.0%	4.1%	20.5%
3+ scores ≤ 25th percentile, or	3.6%	2.7%	13.0%
2+ scores ≤ 16th percentile	5.5%	4.1%	16.4%
**Algorithm B**	**6.4%**	**4.1%**	**18.8%**
5 scores <50th percentile, or	0.9%	0%	7.5%
3+ scores ≤ 25th percentile, or	3.6%	2.7%	13.0%
2+ scores ≤ 16th percentile	5.5%	4.1%	16.4%
**Algorithm C**	**1.8%**	**1.4%**	**12.3%**
5 scores <50th percentile, or	0.9%	0%	7.5%
4+ scores ≤ 25th percentile, or	0%	0%	6.2%
3+ scores ≤ 16th percentile, or	1.8%	1.4%	7.5%
2+ scores ≤ 9th percentile	0.9%	0%	6.5%
**Algorithm D**	**0%**	**0%**	**7.5%**
4+ scores ≤ 25th percentile, or	0%	0%	6.2%
2+ scores ≤ 5th percentile	0%	0%	3.4%

*High Average and Superior categories are based on age-adjusted Crystallized Composite Standard Scores (SS). Bold values designate the base rates for the algorithms (i.e., the frequency at which participants in the normative sample obtained one or more of the performance patterns included the algorithm, whereas non-bolded values reflect the base rate for each specific performance pattern that comprises the algorithm. Algorithm A is a good a priori choice for research and clinical practice for identifying possible mild cognitive impairment in people assumed to have above average or superior premorbid crystallized composite scores (i.e., obtaining a score within that pattern is ~1 SD below the mean). Algorithm B is a good a priori choice for identifying possible impairment in people assumed to have above average or superior premorbid crystallized composite scores, with a greater degree of confidence and a lower false positive rate (i.e., obtaining a score within that pattern is >1.5 SDs below the mean, with a base rate of <7%). Algorithm C is a good a priori choice for identifying possible impairment in people with a university degree, especially if one is not confident in estimating premorbid crystallized composite scores (i.e., obtaining a score within that pattern is >1 SD below the mean). Algorithm D is a good a priori choice for identifying possible impairment in people with a university degree, especially if one is not confident in estimating premorbid crystallized composite scores (i.e., obtaining a score within that pattern is ~1.5 SDs below the mean)*.

Algorithms for identifying cognitive impairment using demographic-adjusted normative scores are presented in Table 4. For Algorithm D, the base rate for people with high average or superior crystalized ability obtaining *any one* of the following, 5 scores <50th percentile, or 3+ scores ≤ 25th percentile, or 2+ scores ≤ 9th percentile, is 6.6 and 5.4%, respectively. As such, a performance pattern on the NIHTB-CB consistent with that algorithm is >1.5 SDs below the mean for people with university degrees.

**Table 4:** Primary algorithms for identifying cognitive impairment in high functioning adults on the NIH toolbox cognition battery using demographic-adjusted normative scores.

	**Crystallized composite**	
	**High average**	**Superior**
	**(*T* = 57–63)**	**(T ≥ 64)**
	**(*n* = 122)**	**(*n* = 56)**
**Algorithms and criteria**	**Base rate**	**Base rate**
**Algorithm A**	**18.9%**	**10.7%**
5 scores <63rd percentile, or	8.2%	0%
4+ scores <50th percentile, or	9.8%	5.4%
3+ scores ≤ 25th percentile, or	3.3%	5.4%
2+ scores ≤ 16th percentile	9.8%	8.9%
**Algorithm B**	**15.6%**	**16.1%**
5 scores <50th percentile, or	1.6%	0%
2+ scores ≤ 25th percentile	14.8%	16.1%
**Algorithm C**	**21.3%**	**16.1%**
5 scores <63rd percentile, or	8.2%	0%
4+ scores <50th percentile, or	9.8%	5.4%
2+ scores ≤ 25th percentile	14.8%	16.1%
**Algorithm D**	**6.6%**	**5.4%**
5 scores <50th percentile, or	1.6%	0%
3+ scores ≤ 25th percentile, or	3.3%	5.4%
2+ scores ≤ 9th percentile	4.1%	0%

*Bold values designate the base rates for the algorithms (i.e., the frequency at which participants in the normative sample obtained one or more of the performance patterns included in the algorithm, whereas non-bolded values reflect the base rate for each specific performance pattern that comprises the algorithm*.

## Discussion

**Figure 1:**
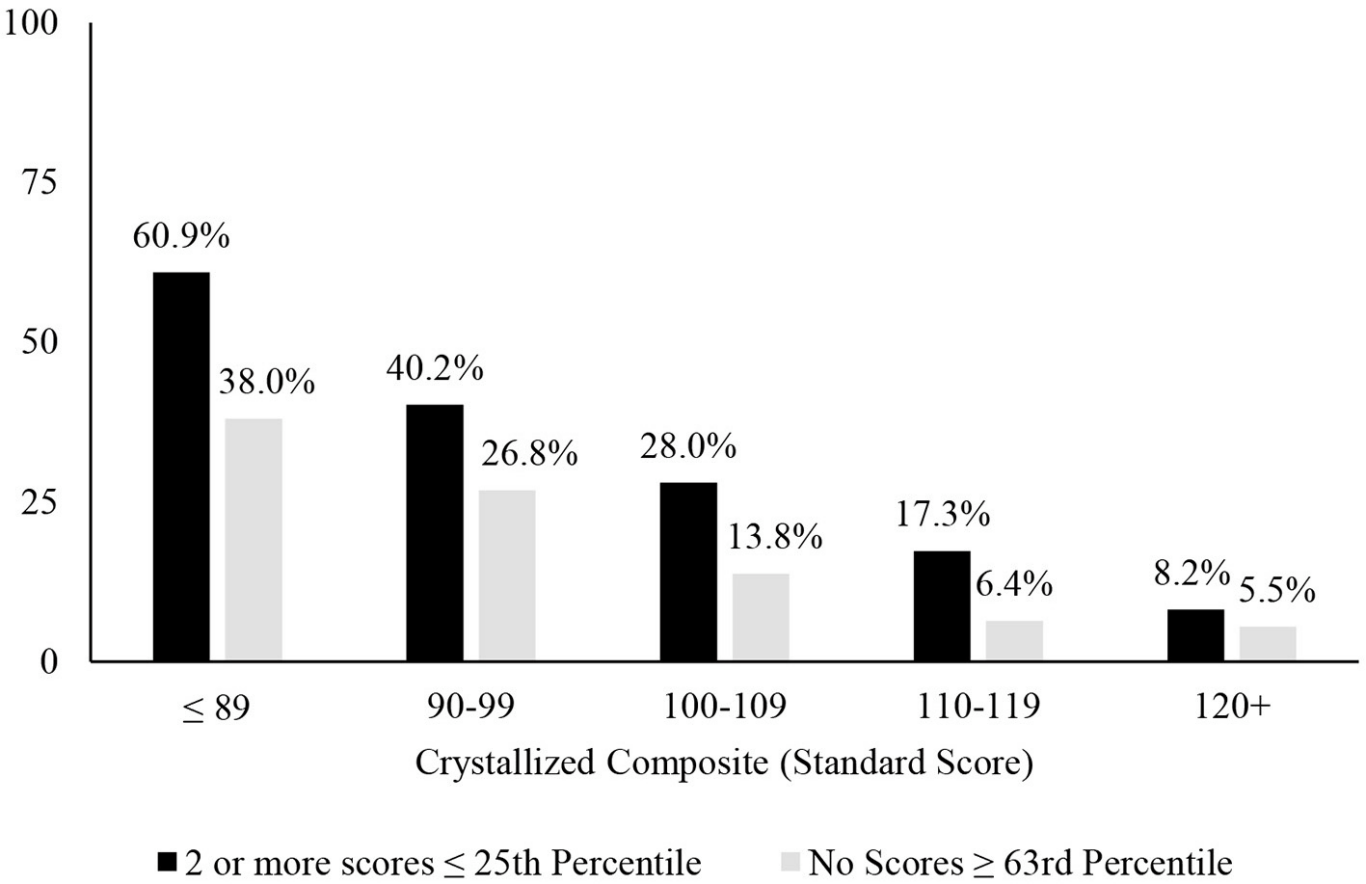
Association between level of intellectual ability and patterns of scores. Percentages of people showing the pattern of scores stratified by their level of intellectual functioning. It is common for people with below average intellectual abilities to have 2 or more fluid scores ≤ 25th and uncommon for people with superior intellectual abilities to have 2 or more below average fluid test scores. Similarly, it is common for people with below average intellectual abilities to have no fluid test scores ≥63rd percentile and it is very uncommon for people with above average or superior intellectual abilities to have no fluid test scores ≥63rd percentile.

A longstanding approach to identifying cognitive deficits, or mild cognitive impairment, is to select a cutoff for defining a low score and applying that cutoff to all people—such as scoring 1 SD (Taylor and Heaton, 2001; Busse et al., 2006) or 1.5 SDs (Lopez et al., 2006; Tabert et al., 2006) below the mean. This approach underlies many studies relating to mild cognitive impairment in older adults (Jak et al., 2009; Ganguli et al., 2011; Petersen et al., 2014; Weissberger et al., 2017) and for identifying mild neurocognitive disorder according to the DSM-5 (American Psychiatric Association, 2013). This approach is also common in clinical practice. A *one-size fits all* approach to identifying cognitive deficits, however, is not appropriate because there are major individual differences in cognitive abilities that must be considered when defining a deficit or impairment, especially a person's level of intellectual functioning and educational history. People with below average intellectual functioning are expected to obtain a large number of low neuropsychological test scores and people with above average or superior intellectual functioning obtain far fewer low test scores (Binder et al., 2009; Brooks et al., 2009b, 2011). This was also true in the present study, as seen in base rates of age-adjusted low scores presented in Table 1, Figure 1. Each year of education corresponds to a one to five point increase in IQ score (Ritchie and Tucker-Drob, 2018), so that people with higher levels of education are expected to obtain fewer low neuropsychological test scores (Brooks et al., 2013). This, too, was illustrated in the present study, as seen in Table 1, whereby those with more years of education also obtained fewer age-adjusted low scores. These individual differences in education and intellectual functioning can only be partially mitigated by using demographic-adjusted normative data, which adjust for education. The differences in base rates of low scores across levels of intelligence were smaller when using demographic-adjusted normative scores compared to age-adjusted normative scores, but still present.

Tables 1, 2 allow clinicians and researchers to determine how common it is to have low scores and high scores on the NIHTB-CB. The base rates of low scores presented in this article differ from those previously published (Holdnack et al., 2017) because we excluded people with health conditions that might have an adverse effect on cognition and Holdnack and colleagues collapsed those with high average and superior crystallized composite scores into a single group. The high score base rate tables presented in this article differ modestly from those previously published (Karr and Iverson, 2020), because participants with neurodevelopmental, psychiatric, substance use, and neurological disorders were excluded in the current analyses, but were included in the prior study.

The algorithms provided in Tables 3, 4 are ready to be applied in clinical studies. Researchers and clinicians should be aware that when using base rate analyses, in research and clinical practice, if multiple algorithms are applied sequentially or simultaneously the base rates increase. For Algorithm A in Table 3, for example, when applying *each* component of the algorithm the base rates range from 2.7 to 5.5%, but when applying *all* components, the base rate is 11.0% in people with superior intellectual abilities.

### Limitations

There are limitations associated with using the NIHTB-CB for identifying cognitive weaknesses, deficits, or impairments. First, the battery is relatively brief. Second, it includes brief measures for some important constructs, such as memory, that lack process-oriented test scores often used to identify different dementias. Although the NIHTB-CB does include an auditory verbal learning test as a supplementary measure, the normative data for this measure is very limited, and does not have the demographic adjustments automatically applied to the core seven tests (Casaletto et al., 2015). Finally, those in the normative sample did not undergo effort testing during the standardization of the battery, meaning that if there were participants with low effort on testing, they could not be identified.

It is important to appreciate, in clinical practice and research, that we used the Crystallized Composite score as an estimate of longstanding intellectual abilities. If research participants have a neurological disorder, or they have sustained a moderate-severe traumatic brain injury, their Crystallized Composite score might underestimate their longstanding, premorbid, intellectual functioning. This is only problematic for our algorithms if the under-estimate results in a *change* in the person's estimated premorbid intellectual *category*—such as moving from high average to average. The differences in base rates between those with estimated superior abilities vs. high average abilities are modest. The real potential problem is for examinees who obtain an age-adjusted Crystallized Composite score between 106 and 109, for example, and the researcher or clinician has good reason to suspect that their longstanding premorbid composite score was likely to be 110 or higher. Research is needed to determine if a small upward adjustment in obtained Crystallized Composite scores, for people who score a few points lower than the high average classification range, improves the diagnostic accuracy of these algorithms in people with neurological conditions.

### Conclusions

In conclusion, the identification of mild cognitive deficits in high-functioning people is challenging in clinical practice and research. High-functioning people are less likely to obtain low neuropsychological test scores than people of average intelligence (Brooks et al., 2011, 2013; Holdnack et al., 2017; Karr et al., 2017, 2018). It is possible that some high functioning people with psychiatric or neurological disorders might not obtain any low scores within a cognitive domain, and if so, it might be the absence of above average scores, not the presence of low scores, that reveals their cognitive deficits. Future research is needed to determine whether a cognitive impairment classification based on these algorithms corresponds to risk factors for, or biomarkers of, clinical conditions known to affect cognitive functioning.

## Data Availability Statement

Publicly available datasets were analyzed in this study. This data can be found at: https://doi.org/10.7910/DVN/FF4DI7.

## Ethics Statement

The secondary analyses of these deidentified data were deemed not human subjects research and were approved by the Partners Human Research Committee (Protocol #: 2020P000504). Written informed consent for participation was not required for this study in accordance with the national legislation and the institutional requirements.

## Author Contributions

GI conceptualized the study, assisted with the literature review, helped conceptualize the analyses, drafted sections of the manuscript, edited the manuscript, and approved the final manuscript. JK assisted with the literature review, helped conceptualize the analyses, conducted the analyses, drafted sections of the manuscript, edited the manuscript, and approved the final manuscript. Both authors contributed to the article and approved the submitted version.

## Funding

GI acknowledges philanthropic support from the Third Option Foundation and the Spaulding Research Institute. The above mentioned entities were not involved in the study design, interpretation of data, the writing of this article, or the decision to submit it for publication.

## Conflict of Interest

GI has received research support from test publishing companies in the past, including PAR, Inc., ImPACT Applications, Inc., and CNS Vital Signs. He receives royalties for one neuropsychological test (Wisconsin Card Sorting Test-64 Card Version). He serves as a scientific advisor for NanoDx®, Sway Operations, LLC, and Highmark, Inc. He has a clinical and consulting practice in forensic neuropsychology, including expert testimony, involving individuals who have sustained mild TBIs. He has received research funding from several test publishing companies, including ImPACT Applications, Inc., CNS Vital Signs, and Psychological Assessment Resources (PAR, Inc.). He has received research funding as a principal investigator from the National Football League, and subcontract grant funding as a collaborator from the Harvard Integrated Program to Protect and Improve the Health of National Football League Players Association Members. He acknowledges unrestricted philanthropic support from ImPACT Applications, Inc., the Mooney-Reed Charitable Foundation, and the National Rugby League. The remaining author declares that the research was conducted in the absence of any commercial or financial relationships that could be construed as a potential conflict of interest.

## Publisher's Note

All claims expressed in this article are solely those of the authors and do not necessarily represent those of their affiliated organizations, or those of the publisher, the editors and the reviewers. Any product that may be evaluated in this article, or claim that may be made by its manufacturer, is not guaranteed or endorsed by the publisher.
